# Induction of CYP3A activity by dexamethasone may not be strong, even at high doses: insights from a case of tacrolimus co-administration

**DOI:** 10.1186/s40780-023-00310-0

**Published:** 2023-12-04

**Authors:** Yoshiyuki Ohno, Toyohito Oriyama, Akira Honda, Mineo Kurokawa, Tappei Takada

**Affiliations:** 1 Department of Pharmacy, The University of Tokyo Hospital, Faculty of Medicine, The University of Tokyo, 7–3-1 Hongo, Tokyo, Bunkyo-Ku 113–8654 Japan; 2 Department of Hematology and Oncology, Graduate School of Medicine, The University of Tokyo, 7–3-1 Hongo, Tokyo, Bunkyo-Ku 113–8654 Japan; 3 Department of Cell Therapy and Transplantation Medicine, The University of Tokyo Hospital, 7–3-1 Hongo, Tokyo, Bunkyo-Ku 113–8654 Japan

**Keywords:** Dexamethasone, CYP3A activity induction, Tacrolimus

## Abstract

**Background:**

Dexamethasone (DEX) induces CYP3A activity in a concentration-dependent manner. However, no study has examined changes in the blood concentration of CYP3A substrate drugs when DEX is administered at high doses. Herein, we present a case in which tacrolimus (TAC), a typical CYP3A substrate drug, was co-administered with a chemotherapy regimen that included high-dose DEX.

**Case presentation:**

A 71-year-old woman underwent liver transplantation for hepatocellular carcinoma 18 years prior to her inclusion in this case study. She was receiving TAC orally at 2 mg/day and had a stable trough blood concentration of approximately 4 ng/mL and a trough blood concentration/dose (C/D) ratio of approximately 2. The patient was diagnosed with post-transplant lymphoproliferative disease (histological type: Burkitt's lymphoma) after admission. Thereafter, the patient received cyclophosphamide-prednisolone (CP), followed by two courses of R-HyperCVAD (rituximab, cyclophosphamide, doxorubicin, vincristine, and DEX) and R-MA (rituximab, methotrexate, and cytarabine) replacement therapy. DEX (33 mg/day) was administered intravenously on days 1–4 and days 11–14 of R-HyperCVAD treatment, and aprepitant (APR) was administered on days 1–5 in both courses. The TAC C/D ratio decreased to approximately 1 on day 11 during both courses, and then increased. Furthermore, a decreasing trend in the TAC C/D ratio was observed after R-MA therapy. The decrease in the TAC C/D ratio was attributed to APR administration rather than to DEX.

**Conclusion:**

The induction of CYP3A activity by a high dose of DEX may not be strong. The pharmacokinetic information on DEX and in vitro enzyme activity induction studies also suggested that CYP3A activity induction is not prominent under high-dose DEX treatment.

## Background

Dexamethasone (DEX) is a steroid used to treat various diseases, and its dosage and duration of use vary widely depending on the indication and purpose for prescription [1]. DEX reportedly induces cytochrome P450 (CYP) 3A activity in vitro in a concentration-dependent manner, [2] and its label describes interactions with CYP3A substrate drugs [3, 4]. CYP3A is a major metabolizing enzyme in the human liver and intestine, and is involved in the metabolism of over half of all marketed drugs [5]. There are two types of clinically important drug–drug interactions (DDIs) [6]. One type is mediated by the inhibition of metabolic enzyme and transporter activities, whereas the other is mediated by the induction of these protein activities [6]. The therapeutic and adverse effects of drugs are often intensified by an increase in their blood concentrations caused by inhibition-based DDIs; however, these effects are generally diminished by a decrease in the blood concentrations caused by induction-based DDIs. Therefore, it is important to assess the extent of induction of CYP3A activity by DEX to ensure that the therapeutic effects of the combination drugs are maintained during co-administration.

McCune et al. reported that the increase in CYP3A activity, determined by measuring testosterone 6-β-hydroxylation level, in human hepatocytes treated with 2, 10, 50, 100, and 250 µM DEX was 1.7-, 1.9-, 3.9-, 6.9-, and 6.6-fold, respectively [2]. This finding indicates that DEX exerts a concentration-dependent effect on CYP3A activity induction [2]. In a study in which triazolam, a CYP3A substrate drug, was administered following low-dose oral DEX (1.5 mg/day) administration, no significant decrease in the blood concentration of triazolam was observed [7]. In an erythromycin breath test conducted on 12 healthy adults, 5-day oral administration of medium-dose DEX (16 mg/day) reportedly increased CYP3A activity by 25.7% [2]. However, the range of increase varied from -8% to 70%, indicating significant individual differences. In addition, the CYP3A activity increased by an average of 55% in patients undergoing the erythromycin breath test 2–9 days after the oral administration of 16–24 mg/day DEX [8]. However, it is important to note that this study included a limited number of participants, with a total of just five patients. Among them, 2 patients received 16 mg/day oral DEX, 1 received 18 mg/day oral DEX, 1 received 24 mg/day oral DEX, and 1 received 175 mg/day intravenous hydrocortisone for 2 days. However, the specific values were not provided for each participant.

DEX is often used at high doses (20–40 mg) for the treatment of multiple myeloma and in palliative care settings [1]. No study has examined changes in the blood levels of CYP3A substrate drugs when DEX is administered at high doses, and the impact of these changes remains unclear. Herein, we report a case in which tacrolimus (TAC), a typical CYP3A substrate drug, was combined with a chemotherapy regimen including high-dose DEX. We discuss the effects of this combination on the blood concentration of TAC and the strength of high-dose DEX in terms of its effect to induce CYP3A activity.

## Case presentation

**Fig. 1:**
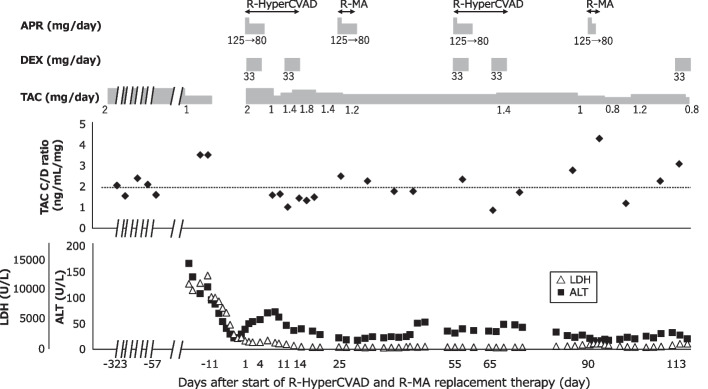
Clinical course of the present patient. The doses of APR, DEX and TAC; TAC C/D ratio; and laboratory parameters are shown. APR, aprepitant; DEX, dexamethasone; TAC, tacrolimus; TAC C/D ratio: TAC trough blood concentration/dose ratio; LDH, lactate dehydrogenase; ALT, alanine aminotransferase

A 71-year-old Japanese woman underwent a living-donor liver transplant for hepatocellular carcinoma 18 years prior to her inclusion in this case study. She was receiving TAC orally at 2 mg/day (twice daily), with a trough blood concentration of approximately 4 ng/mL and stable trough blood concentration/dose (C/D) ratio (ng/mL/mg) of approximately 2. The patient presented night sweats, anorexia, and swelling on the left side of the neck. Owing to the presence of atypical cells in her peripheral blood and a substantial decrease in her platelet count (20,000/µL), she was urgently referred to our hospital for admission, thorough examination, and treatment. The patient was diagnosed with post-transplant lymphoproliferative disease (histological type: Burkitt's lymphoma) after admission. She was managed in the intensive care unit with continuous hemodiafiltration (CHDF) and positive airway pressure (CPAP) for 2 weeks because of tumor collapse syndrome, left subdural hematoma, and acute kidney injury (AKI). Figure 1 illustrates the course of this case. Upon admission, the patient presented with elevated lactate dehydrogenase (LDH; 11,232 U/L) and alanine aminotransferase (ALT; 164 U/L) levels, indicating potential liver dysfunction. Consequently, the TAC dose was reduced from 2 to 1 mg/day. Furthermore, owing to the presence of AKI, TAC administration was temporarily discontinued. As oxygenation improved and the patient's condition progressed, cyclophosphamide–prednisolone (CP) therapy was initiated. Following CP therapy, the LDH and ALT levels decreased. Subsequently, TAC administration was resumed at 2 mg/day. The patient received two cycles of R-HyperCVAD (rituximab, cyclophosphamide, doxorubicin, vincristine, and DEX) and R-MA (rituximab, methotrexate, cytarabine, and methylprednisolone) alternating therapy. DEX (33 mg/day) was administered intravenously on days 1–4 and days 11–14 of the R-HyperCVAD course. Additionally, aprepitant (APR) was administered on days 1–5 in both courses. The TAC dosage was adjusted to maintain a trough blood concentration of approximately 2 ng/mL. Fluconazole 200 mg/day was administered orally throughout the TAC-administration period.

The C/D ratio of TAC during the first course of R-HyperCVAD was initially low (1.07) on day 11, but then increased, reaching 2.57 on day 25. Similarly, during the second course of R-HyperCVAD, the C/D ratio on day 3 (in Fig. 1, day 57) was 2.42, but subsequently decreased to 0.92 on day 11. However, it then exhibited an upward trend, reaching 1.8 on day 18 and 2.86 on day 32. The C/D ratio of TAC on day 4 (in Fig. 1, day 93) of the second course of R-MA was 4.4, but decreased to 1.25 on day 11. Notably, the C/D ratio started to increase during DEX administration on days 11–14 of R-HyperCVAD treatment and did not decrease after 2 weeks, whereas it exhibited a decreasing trend during R-MA treatment. Therefore, the decrease in the C/D ratio was primarily attributed to APR rather than to DEX.

## Discussion and conclusions

TAC is a well-known CYP3A substrate, and its concentration can be influenced by both function of the liver and presence of CYP3A inhibitors and inducers [9]. Additionally, TAC is a P-glycoprotein (P-gp) substrate and may therefore be affected by P-gp inhibitors/inducers [10]. In our case, no significant changes were observed in liver function following the initiation of DEX treatment. In addition to DEX, concomitant medications such as APR and fluconazole can influence CYP3A activity. The patient consistently took 200 mg/day fluconazole while on TAC. Fluconazole is indeed classified as a moderate CYP3A inhibitor [6]. However, considering that the patient was taking the medication concurrently for an extended period, the impact of any fluctuations in TAC concentration was expected to be minimal. APR exhibits both inhibitory and inductive effects on CYP3A activity. In a study involving 12 healthy adults, oral administration of APR at 125 mg on day 1 and 80 mg on days 2–3 was followed by intravenous administration of 2 mg midazolam on days 4, 8, and 15. The area under the blood concentration curve (AUC) ratio for midazolam was 1.25 on day 4, 0.81 on day 8, and 0.96 on day 15 [11]. As these data were obtained after intravenous midazolam administration, it is expected that both inhibition and induction would be affected more when midazolam is administered orally because of the influence of the first-pass effect. APR is also an inducer of CYP2C9 [11], and Ohno et al. reported that its induction effect was observed approximately 2 weeks after the start of APR administration and generally recovered after 3 weeks [12, 13]. The mechanisms of induction of CYP3A and CYP2C activities are generally considered to be similar, and the duration of their induction depends on the turnover rate of the CYP enzymes [14, 15]. In the present case, the TAC C/D ratio decreased approximately 2 weeks after APR administration, with both R-HyperCVAD and R-MA treatments, and then began to increase. These findings suggest that the variation in the TAC C/D ratio in the present case can be attributed mainly to the CYP3A activity-inducing effect of APR. The possibility that the induction effect of DEX also contributes to this phenomenon cannot be ruled out. However, the fact that the TAC C/D ratio increased during DEX administration on days 11–14 of R-HyperCVAD treatment suggests that the effect, if any, is not substantial. Using Horn et al.'s Drug Interaction Probability Scale (DIPS), the interaction with DEX was rated as doubtful. However, the interaction with APR was rated as probable [16].

This case study had some limitations. Induction of CYP activity by DEX is mediated by the pregnane X receptor (PXR/NR1I2), whereas *PXR*/*NR1I2* polymorphisms have been reported to affect DDIs of steroids and TAC [17]. Additionally, *CYP3A5* polymorphisms are known to influence the pharmacokinetics of TAC [10, 18]. These findings imply that the extent of CYP activity induction by DEX may vary depending on the genetic background; however, information regarding these genetic polymorphisms was not available in this case.

Steroids other than DEX used in this case were methylprednisolone (40 mg/day on days 2–4) during R-MA therapy and methylprednisolone 2 mg/day throughout the duration of TAC administration, but the former was only administered for 3 days and the latter at a lower dose. Therefore, the effect on TAC concentration was considered to be minimal.

Doxorubicin and vincristine, administered in this case as part of the chemotherapy regimen, have been reported to be substrates for CYP3A and P-gp in in vitro studies [19–21]. Doxorubicin has also been reported to mildly increase the AUC of docetaxel, a substrate for both CYP3A4 and P-gp, by 50–75% [22]. On the contrary, in a study comprising nine patients treated with chemotherapy regimens containing doxorubicin or vincristine in combination with verapamil, a typical substrate for both CYP3A4 and P-gp, a decrease in AUC was reported in all but one patient, which might have been due to gastrointestinal mucosal damage [23]. No other drug interaction trials have been conducted with these anticancer drugs and CYP3A or P-gp substrate probe. Hence, the impact of these anticancer agents as CYP3A/P-gp inhibitors on the in vivo pharmacokinetics of tacrolimus remains unknown.

Furthermore, although hematocrit levels and inflammatory responses are known to influence the pharmacokinetics of TAC [24–26], no association was observed between the C/D ratio of TAC and the course of hematocrit and C-reactive protein levels (an inflammatory response marker) in the present case.

Recently, Hibino et al. reported that CYP3A activity increases on days 4–8 in patients taking DEX and APR [27]. As mentioned earlier, APR has both CYP3A activity-inhibitory and -inducing effects. In an interaction study on midazolam, the AUC of midazolam increased on day 4 and decreased on day 8 [11]. Therefore, the relatively early increase in CYP3A activity reported by Hibino et al. [27] may be partially attributed to DEX. On the contrary, one of the possible reasons for the increase in TCR C/D to 4.4 on day 4 of the second course of R-MA therapy in the present case may be that inhibition was more significant than induction, as APR was administered for only 3 days.

McCune et al. reported the dose-dependent induction of CYP3A activity in human hepatocytes treated with 2–250 µM DEX; however, CYP3A activity-inducing effect was not observed at levels below 1 µM. Considering that the average blood concentration of DEX at 0.5–3.0 h after 16 mg/day oral administration is 0.1 µM, [2] the blood concentration of DEX intravenously administered at 33 mg/day (oral bioavailability of approximately 80% [28], equivalent to 40 mg/day orally) would be approximately 0.25 µM. Moreover, the concentration of the unbound form would be even lower, which is consistent with the fact that the CYP3A activity-inducing effect was not strong in this case.

Although DEX at moderate doses has been reported to result in mild induction of CYP3A activity in erythromycin breath tests, these reports are highly variable [2, 8]. Moreover, no clinical trial has examined the effect of moderate or higher doses of DEX on blood concentrations of typical CYP3A substrate drugs, and conducting such a trial in the future would be ethically unlikely. Therefore, this case, in which high-dose DEX was combined with TAC, out of clinical necessity, is valuable as it suggests that the CYP3A activity-inducing effect of high-dose DEX is not significant.

In conclusion, the C/D ratio trend of TAC in this case suggested that the CYP3A activity-inducing effect of high-dose DEX was not strong. The pharmacokinetic information on DEX and the results of an in vitro enzyme activity-induction study also support the notion that the CYP3A activity-inducing effect of high-dose DEX is not substantial.

## Data Availability

Data used in this case study will not be shared owing to the risk of identifying the patient.

## Abbreviations

DEX: Dexamethasone
TAC: Tacrolimus
C/D: Trough blood concentration/dose
APR: Aprepitant
CYP: Cytochrome P450
P-gp: P-glycoprotein
LDH: Lactate dehydrogenase
ALT: Alanine aminotransferase
